# Current News

**Published:** 1902-06

**Authors:** 


### Current News.

### BILLS BEFORE THE SENATE, FIFTY-SEVENTH CONGRESS.

Two bills have been presented to the Senate of the United States, April 24,—No. 5419, to add a corps of dental surgeons to the Bureau of Medicine and Surgery of the navy, and 5420, to reorganize the corps of dental surgeons attached to the Medical Department of the army. Both bills were read twice, and the first was referred to the Committee on Naval Affairs and the second to the Committee on Military Affairs. These bills are exactly the same as presented in the House of Representatives, and both approved by the Surgeon-General of the Navy and the Surgeon-General of the Army.

"A BILL TO ADD A CORPS OF DENTAL SURGEONS TO THE BUREAU OF MEDICINE AND SURGERY OF THE NAVY.

"*Be it enacted by the Senate and House of Representatives of the United States of America in Congress assembled,* That to the Bureau of Medicine and Surgery of the Navy there shall be attached a corps of dental surgeons, which corps shall not exceed in number the actual requirements nor the proportion of one to one thousand authorized by law for the naval and marine military service and training schools.

"The said dental corps shall consist of three grades, designated assistant dental surgeon, passed assistant dental surgeon, and dental surgeon, and with respect to rank, pay, and allowances and to promotion within said dental corps the grades named shall correspond to the grades of the medical corps, designated assistant surgeon, passed assistant surgeon, and surgeon, respectively.

"SEC. 2. That original appointments shall be made to the grade of assistant dental surgeon, and the appointees must be citizens of the United States between twenty-one and thirty years of age, graduates of standard dental colleges, of good moral character, of unquestionable professional repute, and shall be required to pass the usual physical examination and a professional examination, which shall include tests of skill and proficiency in practical dentistry and the usual subjects of a standard dental college course: *Provided,* That there shall be first selected a member of the dental profession who is a citizen of the United States and a graduate of a standard dental college and whose aptitude and experience evidence eminent fitness for conducting the professional examinations and for assisting in organizing, equipping, and supervising the operations of the others, who shall be first appointed to the grade of dental surgeon: *Provided, further,* That the dentist now employed at the Naval Academy shall not be displaced by the operation of this Act."

"A BILL TO REORGANIZE THE CORPS OF DENTAL SURGEONS ATTACHED TO THE MEDICAL DEPARTMENT OF THE ARMY.

"*Be it enacted by the Senate and House of Representatives of the United States of America in Congress assembled,* That to the Medical Department of the Army there shall be attached a corps of dental surgeons, which corps shall not exceed in number the actual requirements nor the proportion of one to one thousand authorized by law for service in the Regular Army.

"The said dental corps shall consist of three grades, designated assistant dental surgeon, passed assistant dental surgeon, and dental surgeon, and with respect to rank, pay, and allowances and to promotion within said dental corps the grades named shall correspond to the grades of the medical corps, designated assistant surgeon, passed assistant surgeon, and surgeon, respectively.

"SEC. 2. That original appointments shall be made to the grade of assistant dental surgeon, and the appointees must be citizens of the United States between twenty-two and twenty-nine years of age, graduates of standard dental colleges, of good moral character, of unquestionable professional repute, and shall be required to pass the usual physical examination and a professional examination which shall include tests of skill and proficiency in practical dentistry and the usual subjects of a standard dental college course: *Provided,* That contract dental surgeons attached to the Medical Department of the Army at the time of the passage of this Act may be appointed, three of them to the grade of passed assistant dental surgeon and the others to the grade of assistant dental surgeon."

---

### AMERICAN DENTAL SOCIETY OF EUROPE.

THE next meeting of this society will be held in Stockholm, August 12 to 15, inclusive.

A cordial invitation is extended to the profession to meet with us.

This date will enable those attending the National at Niagara, July 28 to 31, to be present by sailing *via* Hamburg after that meeting.

With the view of facilitating matters for those who propose attending this meeting of the American Dental Society of Europe, I beg to append the following: Owing to the heavy booking of steamer berths, it would be well for intending voyagers to secure their return passages in advance. The best way to reach Stockholm is *via* Hamburg.

1. Travel tickets only for the journey: Hamburg, Kiel, Corsor, Copenhagen, Malmo, Stockholm; returning to Hamburg by the same route, £6 11*s.* 3*d.* per adult, first class; £5 5*s.* 3*d.* per adult, second class.

2. Travel tickets only for the route: Hamburg, Lubeck, and steamer direct to Stockholm, returning to Hamburg by the same route, £4 4*s.* 3*d.* per adult, first class; £3 4*s.* 9*d.* per adult, second class.

In the case of route No. 1, the validity is forty-five days, and for route No. 2, the season. As much notice as possible should be given to secure accommodation. The times between Hamburg and Stockholm are as follows:

Route No. 1: Depart Hamburg, 8.53 A.M. or 11.07 P.M.; arrive Copenhagen, 6.54 P.M. or 10.05 A.M.; depart Copenhagen, 7.45 P.M. or 11.15 A.M.; arrive Stockholm, 11.25 A.M. or 6.45 A.M.

Route No. 2: Depart Hamburg, 12 noon, 2 P.M., or 3.40 P.M.; arrive Lubeck, 1.21 P.M., 3.32 P.M., or 4.53 P.M.; depart Lubeck, about 6.15 P.M., Wednesdays and Saturdays, occupying about forty-two hours, but times for the coming season not yet fixed.

Any further information that may be desired could be obtained from Messrs. Cook & Sons, 261, 262 Broadway, New York.

L. J. MITCHELL,
*Honorary Secretary.*

39 UPPER BROOK STREET, LONDON, W.

---

### NEW JERSEY STATE DENTAL SOCIETY.

### COMMITTEE ON ART AND INVENTION OF THE NEW JERSEY STATE DENTAL SOCIETY.

To all those who during the past year have invented or designed any instrument, appliance, method, or operation in or applicable to the art and science of dental surgery.

The New Jersey State Dental Society respectfully solicits you to send a contribution of such article or appliance, that you have invented or designed, with full description of the same.

All appliances will be classified and receive due consideration at the hands of the Society. We only stipulate that all articles sent shall be of practical value and of general interest to the profession at large.

We will make an interesting exhibit under the head of Art and Invention; one that will be of value not only to the profession, but also to the inventors and designers.

A full report will be made and printed in the Society proceedings.

Send contributions by June 24, and not later than July 1. Otherwise they may not receive proper classification.

All appliances will be well taken care of and returned to the contributors after the session of the Society, which will be held in the Auditorium at Asbury Park, New Jersey, July 16, 17, and 18, 1902.

This year's session will be one of the largest, if not the largest, both in interest and attendance, of any previous session of the New Jersey State Dental Society, which is known for its interesting, valuable, and well-attended sessions.

W. G. Chase,
*Chairman.*

1018 Witherspoon Building, Philadelphia, Pa.

---

### NATIONAL DENTAL ASSOCIATION

In accordance with the result of the recent postal-card vote, the date of the coming meeting of the National Dental Association will be changed from the first Tuesday of August to Monday, July 28, and will continue four days.

A. H. Peck,
*Recording Secretary.*

---

### NORTH CAROLINA STATE BOARD OF DENTAL EXAMINERS.

The North Carolina State Board of Dental Examiners will meet Monday, Tuesday, and Wednesday, June 16, 17, and 18, 1902, at Raleigh, N. C. For further information write the undersigned.

R. H. Jones,
*Secretary.*

Winston-Salem, N. C.

### NATIONAL ASSOCIATION OF DENTAL FACULTIES.

The nineteenth annual convention of the National Association of Dental Faculties will convene in the ball-room of the International Hotel, Niagara Falls, New York, July 24 next. The Executive Committee will meet at eleven A.M., July 23.

All colleges are respectfully referred to the rule requiring that their annual announcement be in the hands of the Executive Committee at this meeting.

H. B. Tileston,
*President.*
S. W. Foster,
*Secretary Executive Committee, N. A. D. F.*

---

### NEW ENGLAND ASSOCIATION OF DENTAL EXAMINERS.

The sixth annual meeting and banquet of the New England Association of Dental Examiners was held at Hotel Brunswick, Boston, Mass., April 22, 1902. All but one of the New England States were well represented. Dr. William Carr and Dr. William Jarvie, of the New York board, and Dr. Charles A. Meeker, of the New Jersey board, were guests of the Association. "Methods of conducting Examinations" and "How best to enforce the Dental Laws" were the subjects for discussion.

The officers elected are: President, Dr. D. W. Fellows, of Portland, Me.; Vice-President, Dr. P. J. Heffern, of Pawtucket, R. I.; Recorder, Dr. George A. Maxfield, of Holyoke, Mass.; Chairman of Executive Committee, Dr. John F. Dowsley, of Boston.

---

### NATIONAL ASSOCIATION OF DENTAL EXAMINERS.

The nineteenth annual session will convene at the International Hotel, Niagara Falls, on Friday, July 25, at ten A.M., and continue in session until the adjournment.

It is earnestly hoped that this session will see a larger representation of delegates than any heretofore held. Every State is asked to make provision now to send delegates. Niagara Falls is an ideal place for meeting, and the International Hotel is the best, the service and appointments first class, and the rates will be according to location of the room. Rates, from \$3.50 to \$4.50 per day, being a reduction of fifty cents per day from the regular rates. It is expected the usual reduction in railroad fare will be arranged in time.

Additional notice will be given in July journals.

J. Allen Osmun,
*Secretary.*

---

### HARVARD DENTAL ALUMNI ASSOCIATION.

The place of meeting of the Harvard Dental Alumni Association for the thirty-first annual banquet, Monday evening, June 23, 1902, has been changed from Young's Hotel, Boston, to the Harvard Union, Cambridge, Mass.

Waldo E. Boardman,
*Secretary.*

Boston, April 22, 1902.

---

### NORTHERN OHIO DENTAL ASSOCIATION.

The forty-third annual meeting of the Northern Ohio Dental Association will be held in Cleveland, Ohio, June 9, 10, and 11, 1902.

W. T. Jackman,
*Corresponding Secretary.*

---

### PENNSYLVANIA STATE DENTAL SOCIETY.

The following are the officers and committees of the Pennsylvania State Dental Society:

President, M. H. Cryer, Philadelphia; First Vice-President, R. H. Nones, Philadelphia; Second Vice-President, J. E. Libbey, Pittsburg; Recording Secretary, C. V. Kratzer, Reading; Corresponding Secretary, V. S. Jones, Bethlehem; Treasurer, R. H. D. Swing, Philadelphia.

*Board of Censors.*—W. D. DeLong, Reading; H. DePuy, J. E. Libbey, J. C. Hertz, C. R. Scholl.

*Executive Committee.*—R. H. Nones, 1708 Chestnut Street, Philadelphia; Grant Mitchell, Pittsburg; H. M. Beck, Wilkes-Barre.

*Legislative Committee.*—G. W. Klump, Williamsport; J. A. Libbey, Pittsburg; A. S. Koser, Harrisburg; H. S. Seip, Allentown; W. E. Van Orsdel, Sharon.

*Committee on Enforcement of Dental Law.*—F. D. Gardiner, Chairman, 1516 Locust Street, Philadelphia; H. Zimmermann, Annville; H. W. Bohn, Reading; D. C. Dunn, Meadville; Herman Haupt, Pittsburg; C. J. Phillips, Pittsburg; C. S. Beck, Wilkes-Barre; C. H. McCowen, West Chester; B. F. Witmer, Lancaster; Robert Huey, Philadelphia; C. C. Walker, Williamsport.

*Clinic Committee.*—J. T. Lippincott, 1427 Walnut Street, Philadelphia; J. E. Libbey, Pittsburg; M. I. Schamberg, Philadelphia.

*Exhibit Committee.*—J. P. Nichol, 124 South Eighteenth Street, Philadelphia; J. C. Hertz, Easton; H. W. Arthur, Pittsburg.

*Publication Committee.*—C. V. Kratzer, Reading; H. B. McFadden, Philadelphia; R. H. D. Swing, Philadelphia.

*Committee on Ethics.*—I. N. Broomell, 1420 Chestnut Street, Philadelphia; J. A. Libbey, Pittsburg; H. E. Register, Philadelphia.

*Committee on Oral Hygiene.*—M. I. Schamberg, 1636 Walnut Street, Philadelphia; W. D. DeLong, Reading; E. W. Bohn, Reading; H. DePuy, Pittsburg; H. N. Young, Wilkes-Barre.

The annual meeting will be held at Bedford Springs, July 8, 9, and 10.

V. S. Jones,
*Corresponding Secretary.*

Bethlehem, Pa.

---

### TENNESSEE DENTAL ASSOCIATION.

The thirty-fifth annual meeting of the Tennessee Dental Association will take place at Mont Eagle, Tenn., beginning Tuesday, July 8, 1902, and continuing three days.

A programme of unusual interest, both as to papers and clinics, has been prepared.

Mont Eagle is a most popular summer resort, so a social as well as a professional treat is in store for those who attend.

The railroads have given a one-and-one-third rate on the certificate plan, and the hotel accommodations are up to date and reasonable.

All ethical dentists are invited to be present and take part in the proceedings.

A. SIDNEY PAGE,
*Secretary.*

COLUMBIA, TENN.

---

### NEW JERSEY STATE DENTAL SOCIETY MEETING.

IN no one profession have there been so many kaleidoscopic changes in methods as in modern dentistry. The practice of to-day is succeeded on the morrow by an improved method. The mechanician, the electrician, the chemist, the microscopist, the histologist, the physician, the biologist, the specialist of the many phases, and the realms of materia medica are all called upon to contribute to the avaricious maw of the present day dentist. The day has passed when the man can sit in his office and read in a desultory way one dental journal, never visit a dental society, and call himself a dentist. The excellence of the professional man is due mostly to the stimulus of the societies; the more meetings he attends the better dentist he is; he must be up and doing and lead a strenuous life; the old order has passed away, the new is on. Show me a community where the societies are progressive and well attended, and the resultant is more men respected professionally and socially, all other things being equal.

A State dental meeting nowadays must be a post-graduate course in dentistry, and in the thirty-odd years of existence the New Jersey State Dental Society has tried to live up to the fact as an argument in dental education and evolution. With this argument emphasized, we ask you, the great body of ethical and progressive men in Jersey and adjacent States, to cut off the week of July 17 and come to our meeting, and let us give you ocular proof of the object we will try and present to you in the wonderful exhibit of 1902, up-to-date electrical appliances, inlay furnaces, chairs, spittoons, improvements in gold and base metals for operative and mechanical work, new and ingenious instruments for the office and laboratory, porcelain teeth and crowns, and last of all clinics (many of them) performed before you by eminent operators that is of more value than a 12mo essay of descriptions.

Asbury Park is a pleasant place to visit. The railroad fares are reasonable, the hotel rates moderate, the scenery is beautiful, and the social element all to be desired.

To the stranger we will try and make the visit pleasant and profitable, and the accomplished members of the entertainment committee will assuage the thirst like the oasis in the desert.

Last year seven hundred dentists registered an attendance; this year we look for even a thousand.

The Columbia will be the head-quarters from which the Hornet's flag flies. Proprietor J. H. Jones will try his best to accommodate all who favor him with the request in a reasonable time, with the rate of $2.50 and $3.50 per day.

The auditorium, the largest building on the Jersey coast, will be used in its entirety for the exhibits and meetings.

Again we ask you to cut the time off now and meet with us.

Charles. A. Meeker, D.D.S.,
*Secretary.*
H. S. Sutphen. D.D.S..
*Assistant Secretary.*

Newark, N. J.

---

### COLORADO STATE DENTAL ASSOCIATION.

The sixteenth annual meeting of the Colorado State Dental Association will be held in the Alta Vista Hotel, Colorado Springs, June 17, 18, and 19, 1902.

A good meeting is assured. Special rates at the hotels. A cordial invitation is extended to the profession.

W. A. Brierly,
*Secretary.*

70 Barth Block, Denver, Col.

